# Antibiotic use and gastrointestinal symptoms among adults in the West Bank, Palestine: A cross-sectional study

**DOI:** 10.1371/journal.pone.0353315

**Published:** 2026-11-13

**Authors:** Joud Abu Zahra, Hamza Joma, Ahmad Awwad, Rabee Radwan, Hana Khalili, Yahya Omran, Faridah Ihmoud

**Affiliations:** 1 Department of Medicine, Faculty of Medicine and Allied Medical Sciences, An-Najah National University, Nablus, Palestine; 2 Department of Scientific Research and Medical Projects, Faculty of Medicine and Allied Medical Sciences, An-Najah National University, Nablus, Palestine; The Chinese University of Hong Kong, HONG KONG

## Abstract

**Background:**

Antibiotics are extensively used to treat infections caused by bacteria and have had a substantial impact on improving public health. However, they can change the composition of the gut microbiota and induce gastrointestinal symptoms including diarrhea, constipation, bloating, abdominal pain and reflux. Although these effects have been described in different populations, evidence from the West Bank remains limited.

**Materials and methods:**

A cross-sectional study was conducted among adults aged 18 years and older in the West Bank. Participants were recruited using non-probability convenience sampling, and data were collected through an online questionnaire disseminated through social media platforms. The questionnaire evaluated demographic characteristics, antibiotic use patterns over the previous three months, self-medication habits, lifestyle factors, and gastrointestinal symptoms. Statistical analyses included descriptive statistics, chi-square tests, Mann–Whitney U tests, and binary logistic and linear regression, with a negative binomial model as a sensitivity analysis.

**Results:**

This study comprised 403 participants from the West Bank (mean age: 34.16 years, 55.3% men). Overall, 47.4% of participants reported antibiotic use over the last three months, and 56.8% reported having clinically relevant GI symptoms. In unadjusted analysis, antibiotic users had significantly higher GI symptom scores than non-users (p  =  0.003), and A positive trend in GI symptom prevalence was observed across antibiotic-course categories (p for trend = 0.027). However, after adjustment for demographic, clinical, and lifestyle factors, antibiotic use was not independently associated with clinically relevant GI symptoms (adjusted OR = 1.06, 95% CI: 0.69–1.65, p = 0.788) or GI symptom score.

**Conclusions:**

In this West Bank sample, recent antibiotic use was not independently associated with gastrointestinal symptoms after adjustment for potential confounders. Greater symptom burden was associated mainly with pre-existing gastrointestinal conditions, psychological stress, and recent infection, suggesting that the crude association may reflect underlying clinical factors rather than antibiotic exposure itself. Prospective studies with detailed antibiotic exposure and temporal data are needed to clarify these relationships.

## Introduction

The use of antibiotics has markedly improved the management of infectious diseases. However, inappropriate dosage or treatment duration can increase the risk of adverse effects. Gastrointestinal symptoms are among the most common antibiotic-related adverse effects. Antibiotic-induced gastrointestinal adverse effects include nausea, bloating, constipation, diarrhea, and stomach pain [1]. These symptoms are primarily attributed to the disruption of the gut microbiota (dysbiosis), a complex community of microorganisms that plays a vital role in digestion, immune regulation, and general health [2,3]. Antibiotic-induced dysbiosis can occur even with short courses of treatment and may persist for weeks, contributing to both acute discomfort and potential long-term consequences [4,5].

Globally, gastrointestinal side effects represent a significant proportion of antibiotic-related adverse events and contribute to increased healthcare burden, including reduced quality of life, additional medical consultations, and, in some cases, discontinuation of therapy [6,7]. Surprisingly, despite the widespread use of antibiotics, public awareness of these side effects, particularly those affecting the gastrointestinal system, remains variable and often limited [8].

In Palestine, antibiotics are widely used and frequently without prescription, which may increase unnecessary exposure and the risk of adverse effects [9]. Local research on the connection between antibiotic usage and the incidence of gastrointestinal problems in the general adult population, as well as the degree of awareness of these symptoms, is, nevertheless, lacking.

Thus, this study aimed to assess gastrointestinal symptom burden among adults in the West Bank and examine its association with recent antibiotic use.

## Materials and methods

### Study design and population

This was a cross-sectional study conducted in the West Bank in 2026 using a structured questionnaire to examine the association between antibiotic use and gastrointestinal symptoms among adults. The study included adults ≥ 18 years old, currently residing in Palestine, and who were able to provide informed consent. Participants who were younger than 18 years old, did not provide informed consent, or who had not completed the questionnaire’s scale items were excluded.

### Sample size

The sample size was calculated using the Raosoft sample size calculator [10]. Using a 95% confidence level and a 5% margin of error with an assumed population proportion of 50%, the minimum effective sample size required was 385. The final sample comprised 403 participants, exceeding the calculated minimum sample size.

### Data collection tools, validity, and reliability

All the data were collected via a structured questionnaire using a non-probability convenience sampling technique. The questionnaire was disseminated via social media platforms using Google Forms. To reduce duplicate participation, the form was restricted to one response per Google account and responses were additionally checked manually for potential duplicates. Most questionnaire items were mandatory, and the estimated time for completion was approximately 5–10 minutes.

The questionnaire consisted of sociodemographic variables (age, gender, place of residence, educational level, marital status, occupational status, monthly income), clinical and lifestyle-related variables (coffee consumption, smoking status, fast food consumption, perceived stress level (assessed with a validated single-item measure [11]), proton pump inhibitor (PPI) use, non-steroidal anti-inflammatory drug (NSAID) / analgesic use, previous GI diagnosis, GI infection within the past 4 weeks, and recent antibiotic use within the last 3 months). Additionally, it consisted of variables regarding antibiotic exposure (number of courses, time since last course, duration of the last course, whether the last antibiotic course was physician-prescribed, adherence to the course, simultaneous use of multiple antibiotic courses, and probiotic recommendation by health-care professionals). Moreover, gastrointestinal symptoms during the past 4 weeks were assessed using a nine-item symptom questionnaire developed for this study, with symptom domains adapted from the conceptual categories of the Gastrointestinal Symptom Rating Scale (GSRS), covering reflux, abdominal pain, indigestion, diarrhea, and constipation domains [12–14]. The study-specific nine-item questionnaire was not the original GSRS instrument and did not undergo formal psychometric validation. Each symptom was rated on a 4-point Likert scale (0 = none, 1 = mild, 2 = moderate, 3 = severe). A composite GI symptom score was calculated by summing the nine items. Symptom frequency and impact on daily activities were also assessed. Participants were asked to report the timing of GI symptom onset relative to their most recent antibiotic course to explore temporal association.

The questionnaire was pilot-tested on a sample of students (n = 20) to assess comprehensibility and face validity, and minor wording modifications were made accordingly. In the present study, the nine-item symptom scale demonstrated good internal consistency (Cronbach’s α = 0.861).

### Ethical approval

The study was approved by the Institutional Review Board (IRB) of An-Najah National University (IRB Reference #: Med. Mar. 2026/52). It is an observational study, and no clinical interventions were performed. The research was conducted in compliance with the Declaration of Helsinki.

The participants were informed about the purpose of the study, and informed consent was obtained before their participation. Confidentiality and privacy were maintained throughout the study, with access to participants’ data restricted to the research team.

### Statistical analysis

The Statistical Package for the Social Sciences (SPSS) version 27 was used to analyze the data. Descriptive statistics were used to summarize the study variables; categorical variables are presented as frequencies and percentages, whereas continuous variables are presented as medians and interquartile ranges (IQRs). The data were not normally distributed, as tested by the Kolmogorov-Smirnov test.

The internal consistency of the nine-item GI symptom scale was assessed using Cronbach’s α. Each item was rated from 0 (none) to 3 (severe), and the total GI symptom score was calculated as the sum of the nine items (range 0–27). For the purposes of this study, clinically relevant GI symptoms were operationally defined as the presence of at least one symptom rated as moderate or severe (score ≥2 on the 0–3 response scale). This threshold was selected to distinguish symptoms of at least moderate perceived severity from absent or mild symptoms and was used as a study-specific binary outcome rather than as a formally validated diagnostic cutoff.

The chi-square test was used to assess associations between categorical variables. The Mann-Whitney U test was used to compare the GI symptom score between antibiotic users and non-users.

Two multivariable models were constructed using an identical set of covariates: a binary logistic regression model for clinically relevant GI symptoms (yes/no) and a multiple linear (ordinary least squares) regression model for the continuous GI symptom score. Both models included antibiotic use, age, gender, smoking, fast-food consumption, perceived stress, recent GI infection, PPI use, NSAID use, and previous GI diagnosis. Fast-food consumption (reference, rarely) and perceived stress (reference, mild) were entered as categorical variables, and “no” or male served as the reference category for the remaining binary predictors. For the logistic model, adjusted odds ratios (AOR) with 95% confidence intervals (CI) were reported, and model performance was evaluated using the omnibus χ² test, the Nagelkerke R², the Hosmer-Lemeshow goodness-of-fit test, and overall classification accuracy. The discriminative ability of the logistic regression model was additionally assessed using receiver operating characteristic (ROC) curve analysis of the model-predicted probabilities, with the area under the curve (AUC) and 95% confidence interval reported.

For the linear model, unstandardized coefficients (B) with 95% CI, standardized coefficients (β), and the model R² and F test were reported. Multicollinearity was assessed using tolerance and variance inflation factors. As a sensitivity analysis, given the count nature of the symptom score, a negative binomial regression model with a log link was fitted using the same covariates, and adjusted rate ratios (aRR) with 95% CI were reported. All tests were two-sided, and p < 0.05 was considered statistically significant.

### Use of artificial intelligence tools

OpenAI’s ChatGPT was used during manuscript preparation for language editing, grammatical correction, and improving the clarity and readability of selected sections of the manuscript. The tool was not used to generate or modify primary research data, perform statistical analyses, determine study outcomes, or independently formulate the study hypotheses, interpretations, or conclusions. All AI-assisted text was critically reviewed and edited by the authors, who verified its accuracy, consistency with the study data, and appropriateness of the cited literature. The authors take full responsibility for the final content of the manuscript.

## Results

### Participant characteristics

A total of 403 participants were included, with no missing data on any analyzed variable. The mean age was 34.16 years (SD 13.26; median 30, IQR 23–43; range 18–71). Slightly more than half were male (223, 55.3%), and most reported a bachelor’s degree or diploma as their highest educational level (321, 79.7%). Just over half resided in a village or refugee camp (229, 56.8%), were married (217, 53.8%), and were employed or running a private business (219, 54.3%). Regarding clinical and lifestyle factors, 117 participants (29.0%) were current smokers, 117 (29.0%) reported a previous gastrointestinal (GI) diagnosis, 69 (17.1%) had experienced a GI infection in the preceding four weeks, 66 (16.4%) used proton-pump inhibitors (PPIs), and 145 (36.0%) used NSAID or other analgesics. Moderate and severe perceived stress were reported by 194 (48.1%) and 110 (27.3%) participants, respectively. The full distribution of sociodemographic, clinical, and lifestyle characteristics is presented in Table 1.

**Table 1:** Participant characteristics and clinical/lifestyle factors (N = 403).

Characteristic	Value
**Age (years)**	
Median (IQR)	30 (23-43)
Mean ± SD	34.16 ± 13.26
Range	18-71
**Gender**	
Male	223 (55.3)
Female	180 (44.7)
**Place of residence**	
City	174 (43.2)
Village/refugee camp	229 (56.8)
**Educational level**	
High school or less	40 (9.9)
Bachelor’s degree or diploma	321 (79.7)
Higher education	42 (10.4)
**Marital status**	
Single	186 (46.2)
Married	217 (53.8)
**Occupational status**	
Student	108 (26.8)
Employed/private business	219 (54.3)
Unemployed/retired	76 (18.9)
**Monthly income (ILS)**	
<1500	112 (27.8)
1500-3000	124 (30.8)
3000-5000	97 (24.1)
>5000	70 (17.4)
**Smoking**	
No	286 (71.0)
Yes	117 (29.0)
**Coffee consumption**	
None	110 (27.3)
One cup	122 (30.3)
2-3 cups	125 (31.0)
>3 cups	46 (11.4)
**Fast food consumption**	
Rarely	239 (59.3)
1-2 times/week	123 (30.5)
≥3 times/week	41 (10.2)
**Perceived stress**	
Mild	99 (24.6)
Moderate	194 (48.1)
Severe	110 (27.3)
**PPI/acid-suppressant use**	
No	337 (83.6)
Yes	66 (16.4)
**NSAID/analgesic use**	
No	258 (64.0)
Yes	145 (36.0)
**Previous GI diagnosis**	
No	286 (71.0)
Yes	117 (29.0)
**GI infection (past 4 weeks)**	
No	334 (82.9)
Yes	69 (17.1)
**Antibiotic use (past 3 months)**	
No	212 (52.6)
Yes	191 (47.4)
**Gastrointestinal symptom outcome**	
GI symptom score, median (IQR)	6 (3-11)
GI symptom score, mean ± SD (range)	6.83 ± 5.54 (0-27)
Clinically relevant GI symptoms: No	174 (43.2)
Clinically relevant GI symptoms: Yes	229 (56.8)

Values are n (%) unless otherwise indicated. GI = gastrointestinal; IQR = interquartile range; SD = standard deviation; PPI = proton-pump inhibitor; NSAID = non-steroidal anti-inflammatory drug. Clinically relevant GI symptoms were defined as at least one symptom rated moderate or severe (score ≥2).

The nine-item GI symptom scale demonstrated good internal consistency (Cronbach’s α = 0.861). The GI symptom score was non-normally distributed (test of normality p < 0.001), with a median of 6 (IQR 3–11; mean 6.83, SD 5.54; range 0–27). Clinically relevant GI symptoms were present in 229 participants (56.8%).

### Antibiotic use patterns

Antibiotic use within the previous three months was reported by 191 participants (47.4%). Among these users (Table 2), most had taken a single course (108, 56.5%), while 16 (8.4%) reported four or more courses. The most recent course had been taken within the previous 1–3 months in 85 users (44.5%) and within the past seven days in 38 (19.9%). The majority of courses had been physician-prescribed (140, 73.3%), and 120 users (62.8%) reported full adherence to the prescribed regimen. Concurrent use of multiple antibiotics was uncommon (26, 13.6%), and only 21 users (11.0%) recalled receiving advice to take a probiotic.

**Table 2:** Antibiotic exposure characteristics among antibiotic users (n = 191).

Characteristic	n (%)
**Number of antibiotic courses**	
1 course	108 (56.5)
2 courses	50 (26.2)
3 courses	17 (8.9)
≥4 courses	16 (8.4)
**Time since most recent course**	
Last 7 days	38 (19.9)
8-30 days	68 (35.6)
1-3 months	85 (44.5)
**Duration of most recent course**	
Do not remember	30 (15.7)
<5 days	67 (35.1)
5-7 days	72 (37.7)
>7 days	22 (11.5)
**Prescribed by a physician**	
No	51 (26.7)
Yes	140 (73.3)
**Adherence to the course**	
No	33 (17.3)
Partly	38 (19.9)
Yes	120 (62.8)
**Simultaneous use of multiple antibiotic courses**	
No	165 (86.4)
Yes	26 (13.6)
**Probiotic advice given**	
No	138 (72.3)
Yes	21 (11.0)
Do not remember	32 (16.8)

Percentages are calculated within antibiotic users (n = 191). Denominators differ from the full sample (N = 403) because these items applied only to participants reporting antibiotic use in the past 3 months.

### Bivariate associations between antibiotic use and gastrointestinal symptoms

In the unadjusted analysis (Table 3), clinically relevant GI symptoms were more frequent among antibiotic users than non-users (118/191, 61.8% vs 111/212, 52.4%), but this difference did not reach statistical significance (Pearson χ² p = 0.057; crude OR 1.47, 95% CI 0.99–2.19). The prevalence of clinically relevant symptoms increased across categories of antibiotic exposure, from 52.4% in non-users to 75.0% in those reporting four or more courses; although the overall association was not significant (Pearson χ² p = 0.221), a significant linear-by-linear trend was observed (p = 0.027).

**Table 3:** Crude association between antibiotic use and gastrointestinal outcomes (N = 403).

Antibiotic use	No clinically relevant GI symptoms, n (%)	Clinically relevant GI symptoms, n (%)	OR (95% CI)	p
**Panel A. Antibiotic use and clinically relevant GI symptoms**				
No (n = 212)	101 (47.6)	111 (52.4)	1.00 (reference)	—
Yes (n = 191)	73 (38.2)	118 (61.8)	1.471 (0.989-2.188)	0.057
**Panel B. Number of antibiotic courses and clinically relevant GI symptoms (dose-response)**				
**Number of antibiotic courses**	**n**	**Clinically relevant GI symptoms, n (%)**		
Non-users	212	111 (52.4)		
1 course	108	65 (60.2)		
2 courses	50	29 (58.0)		
3 courses	17	12 (70.6)		
≥4 courses	16	12 (75.0)		
**Panel C. GI symptom score by antibiotic use**				
**Antibiotic use**	**Median (IQR)**	**Mean ± SD**		
No	5 (2-9)	6.03 ± 5.17		
Yes	7 (3-12)	7.71 ± 5.80		

*Pearson χ² p = 0.221; linear-by-linear association (trend) p = 0.027.*

*Mann-Whitney U = 16758.000, Z = −2.996, p = 0.003. OR = crude odds ratio; CI = confidence interval; GI = gastrointestinal; IQR = interquartile range; SD = standard deviation. Panel A p-value from Pearson χ². GI symptom score was non-normally distributed (normality test p < 0.001); the Mann-Whitney U test was therefore used.*

The GI symptom score was significantly higher among antibiotic users than non-users (median 7, IQR 3−12 vs median 5, IQR 2−9; mean 7.71, SD 5.80 vs mean 6.03, SD 5.17; Mann-Whitney U = 16758.0, Z = −2.996, p = 0.003).

### Multivariable analyses

Two multivariable models were fitted using the same set of covariates to examine the clinically relevant symptom outcome (binary logistic regression; Table 4) and the continuous symptom score (linear regression; Table 5). After adjustment, antibiotic use was not independently associated with either outcome (adjusted OR 1.06, 95% CI 0.69–1.65, p = 0.788; adjusted B = 0.47, 95% CI −0.50 to 1.43, p = 0.341), indicating that the higher crude symptom burden among users was accounted for by other factors.

**Table 4:** Multivariable logistic regression for clinically relevant GI symptoms (N = 403).

Predictor	AOR	95% CI	p
Antibiotic use (yes vs no)	1.062	0.685-1.647	0.788
Age (per 1 year)	0.996	0.978-1.014	0.670
Female (vs male)	0.595	0.362-0.978	**0.041**
Smoking (yes vs no)	1.416	0.835-2.401	0.197
**Fast food consumption (ref: rarely)**			
1-2 times/week	0.876	0.523-1.464	0.613
≥3 times/week	2.047	0.924-4.537	0.078
**Perceived stress (ref: mild)**			
Moderate	2.092	1.227-3.568	**0.007**
Severe	2.534	1.358-4.727	**0.003**
PPI/acid-suppressant use (yes vs no)	2.814	1.366-5.796	**0.005**
NSAID/analgesic use (yes vs no)	1.431	0.910-2.250	0.121
GI infection, past 4 weeks (yes vs no)	1.752	0.957-3.209	0.069
Previous GI diagnosis (yes vs no)	2.497	1.474-4.229	**0.001**

Binary logistic regression; outcome = clinically relevant GI symptoms. N = 403 (no missing data); model converged at iteration 4. Omnibus model χ²(12) = 62.987, p < 0.001; Nagelkerke R² = 0.194; Hosmer-Lemeshow p = 0.443; overall correct classification 67.2%. AOR = adjusted odds ratio; CI = confidence interval; ref = reference category; GI = gastrointestinal; PPI = proton-pump inhibitor; NSAID = non-steroidal anti-inflammatory drug. Bold p-values are significant at p < 0.05.

**Table 5:** Multivariable linear regression for gastrointestinal symptom score (N = 403).

Predictor	B	95% CI	β	p
Antibiotic use (yes vs no)	0.465	−0.495 to 1.426	0.042	0.341
Age (per 1 year)	−0.013	−0.053 to 0.027	−0.032	0.515
Female (vs male)	−1.788	−2.862 to −0.715	−0.161	**0.001**
Smoking (yes vs no)	0.831	−0.303 to 1.965	0.068	0.150
**Fast food consumption (ref: rarely)**				
1-2 times/week	−0.214	−1.340 to 0.912	−0.018	0.709
≥3 times/week	0.666	−1.031 to 2.364	0.036	0.441
**Perceived stress (ref: mild)**				
Moderate	1.670	0.488 to 2.851	0.151	**0.006**
Severe	2.753	1.387 to 4.118	0.222	**<0.001**
PPI/acid-suppressant use (yes vs no)	1.131	−0.280 to 2.541	0.076	0.116
NSAID/analgesic use (yes vs no)	0.808	−0.176 to 1.792	0.070	0.107
GI infection, past 4 weeks (yes vs no)	2.453	1.191 to 3.714	0.167	**<0.001**
Previous GI diagnosis (yes vs no)	4.146	3.031 to 5.262	0.340	**<0.001**
(Constant)	3.958	1.972 to 5.944	—	<0.001

Multivariable linear (OLS) regression; outcome = GI symptom score (range 0–27). N = 403, no missing data. R² = 0.285, adjusted R² = 0.263; model F(12, 390) = 12.965, p < 0.001; Durbin-Watson = 1.833; all VIF ≤ 1.71 (no collinearity concern). Fast food and perceived stress were entered as categorical (dummy) variables using the same reference categories as Table 4 (rarely and mild), so the two adjusted models are directly comparable. B = unstandardized coefficient (adjusted change in GI symptom score relative to the reference); β = standardized coefficient; CI = confidence interval; GI = gastrointestinal; PPI = proton-pump inhibitor; NSAID = non-steroidal anti-inflammatory drug. Bold p-values are significant at p < 0.05.

The two adjusted models identified a consistent set of independent correlates of greater GI symptom burden. A previous GI diagnosis was the strongest correlate in both models (adjusted OR 2.50, 95% CI 1.47–4.23, p = 0.001; adjusted B = 4.15, 95% CI 3.03–5.26, p < 0.001). Higher perceived stress showed a graded association, with both moderate (adjusted OR 2.09, p = 0.007; B = 1.67, p = 0.006) and severe stress (adjusted OR 2.53, p = 0.003; B = 2.75, p < 0.001) associated with greater symptoms relative to mild stress. A recent GI infection was associated with a higher symptom score (adjusted B = 2.45, 95% CI 1.19–3.71, p < 0.001) and showed a borderline association with the binary outcome (adjusted OR 1.75, 95% CI 0.96–3.21, p = 0.069). Female sex was associated with lower symptom burden in both models (adjusted OR 0.60, p = 0.041; B = −1.79, p = 0.001). PPI use was independently associated with clinically relevant symptoms in the logistic model (adjusted OR 2.81, 95% CI 1.37–5.80, p = 0.005) but not with the continuous score (B = 1.13, p = 0.116). Age, smoking, fast-food consumption, and NSAID use were not independently associated with either outcome.

The logistic model was statistically significant (omnibus χ²(12) = 62.99, p < 0.001), showed adequate calibration (Hosmer-Lemeshow p = 0.443), explained 19.4% of the variance (Nagelkerke R²), and correctly classified 67.2% of cases. ROC analysis of the model-predicted probabilities yielded an AUC of 0.715 (95% CI 0.665–0.765, p < 0.001), indicating that the model discriminated between participants with and without clinically relevant GI symptoms better than chance.

The linear model was likewise significant (F(12, 390) = 12.97, p < 0.001) and explained 28.5% of the variance in symptom score (adjusted R² = 0.263), with no evidence of problematic multicollinearity (all variance inflation factors ≤ 1.71).

In a negative binomial sensitivity model (Table 6), which accounts for the count nature of the symptom score, the findings were materially unchanged. Antibiotic use remained unassociated with symptom burden (adjusted rate ratio (aRR) 1.07, 95% CI 0.86–1.34, p = 0.528), whereas a previous GI diagnosis (aRR 1.74, 95% CI 1.36–2.23, p < 0.001), moderate (aRR 1.49, p = 0.004) and severe perceived stress (aRR 1.66, p = 0.001), a recent GI infection (aRR 1.44, p = 0.012), and (inversely) female sex (aRR 0.75, p = 0.022) remained the independent correlates of greater symptom burden.

**Table 6:** Negative binomial regression for gastrointestinal symptom score: sensitivity analysis (N = 403).

Predictor	aRR	95% CI	p
Antibiotic use (yes vs no)	1.07	0.86–1.34	0.528
Age (per 1 year)	1.00	0.99–1.01	0.847
Female (vs male)	0.75	0.59–0.96	**0.022**
Smoking (yes vs no)	1.15	0.88–1.49	0.305
**Fast food consumption (ref: rarely)**			
1–2 times/week	0.94	0.72–1.21	0.609
≥3 times/week	1.10	0.75–1.63	0.619
**Perceived stress (ref: mild)**			
Moderate	1.49	1.13–1.95	**0.004**
Severe	1.66	1.22–2.27	**0.001**
PPI/acid-suppressant use (yes vs no)	1.16	0.85–1.60	0.348
NSAID/analgesic use (yes vs no)	1.20	0.96–1.51	0.101
GI infection, past 4 weeks (yes vs no)	1.44	1.08–1.92	**0.012**
Previous GI diagnosis (yes vs no)	1.74	1.36–2.23	**<0.001**

Negative binomial regression with log link; outcome = GI symptom score (count, range 0–27). N = 403, no missing data. Likelihood-ratio model χ²(12) = 66.33, p < 0.001; deviance/df = 0.83 (no overdispersion); AIC = 2370.2. aRR = adjusted rate ratio (exp B), expressed for each category relative to its reference so that values are directly comparable with Tables 4 and 5; CI = confidence interval; GI = gastrointestinal; PPI = proton-pump inhibitor; NSAID = non-steroidal anti-inflammatory drug. Reference categories: rarely (fast food) and mild (perceived stress). Bold p-values are significant at p < 0.05.

## Discussion

To our knowledge, this is one of the first studies in the West Bank to examine the association between recent antibiotic use and GI symptoms in the general adult population. Although antibiotic users reported a greater GI symptom burden in the crude analysis, antibiotic use was not independently associated with either clinically relevant GI symptoms or continuous GI symptom score after multivariable adjustment.

Antibiotic use within the previous three months was reported by 191 participants. Among these users, 140 (73.3%) reported that their most recent course had been prescribed by a physician, and 120 (62.8%) reported full adherence to the prescribed regimen. This pattern differs from prior Palestinian studies showing that self-medication and non-prescribed antibiotic use are widespread public health concerns, with 98% of university student respondents reporting that they practiced self-medication, with antibiotics accounting for 19.9% of the commonly used medications [9].

Similarly, a study of adults recruited from community pharmacies in Hebron and Bethlehem reported a prevalence of antibiotic self-medication of 76.4%; only 26.1% completed the full antibiotic course [15]. Differences between our findings and those of these two studies may reflect differences in the population. The earlier studies specifically examined self-medication among university students or pharmacy attendees, whereas our study assessed any antibiotic use in the general population. Additionally, our sample consisted of more highly educated participants, which may have contributed to greater reliance on physician-prescribed medications. Prior studies have associated higher educational attainment with more appropriate antibiotic use, including greater reliance on physician-prescribed medication [16]. Higher health literacy has also been associated with better medication adherence [17]. Moreover, social desirability bias may have led to overreporting of physician prescription and adherence.

Only 21 (11.0%) users recalled receiving advice to take a probiotic. This may reflect the limited routine counseling about probiotics during antibiotic prescribing. A study among Palestinian health care professionals reported that pharmacists recommended daily probiotics use significantly more frequently than physicians (20.7% versus 7.5%) [18]. The low proportion of participants who reported receiving advice to take probiotics during antibiotic treatment in our study may reflect the international differences in co-administration practices, as evidenced by a multinational study that has shown varied behaviors regarding probiotics across countries and clinical settings [19].

In the unadjusted analysis, clinically relevant GI symptoms were more frequent among antibiotic users than non-users, although the difference was not statistically significant. Previous studies have linked antibiotic use to the disruption of the gut microbiota and changes in the microbial composition that may contribute to antibiotic-associated diarrhea and altered bowel habits [20,21]. Other studies have associated antibiotic exposure with antibiotic‑associated diarrhea, *Clostridium difficile* infection, and subsequent IBS in some settings [22,23].

However, the adjusted model in our study demonstrated that antibiotic use was not independently associated with GI symptoms. This does not contradict the biological effects of antibiotics. Rather, it suggests that in this sample the crude association was explained by confounding. People who use antibiotics are not randomly selected from the population; they often receive antibiotics because they have infections, inflammation, or other illnesses that may themselves cause GI symptoms. This is known as confounding by indication, a common problem in observational medication studies [24–26]. In our data, this explanation is supported by the fact that recent GI infection and previous GI diagnosis remained important predictors after adjustment, while antibiotic use became non-significant. Thus, antibiotic use may have served as a marker of recent illness or underlying GI vulnerability, potentially contributing to the crude association with current symptoms. However, residual confounding cannot be excluded because the indication for antibiotic treatment, infection severity, detailed dietary exposures, and other comorbidities were not fully measured.

This also explains why our findings may differ from previous studies linking antibiotics to IBS or antibiotic-associated diarrhea. Many prior studies used longitudinal designs, prescription records, or clinically defined outcomes such as IBS, antibiotic-associated diarrhea, or *Clostridioides difficile* infection. In contrast, our study was cross-sectional and measured self-reported antibiotic use within the previous three months together with current non-specific GI symptoms. Antibiotic exposure was self-reported, and we did not have detailed information on the specific antibiotic agent or class, treatment indication, dose, exact timing, or sufficient temporal information to determine whether symptoms began before or after antibiotic use. This is important because gastrointestinal effects may differ substantially according to the specific antibiotic or pharmacological class used. Prior research has demonstrated considerable variation in the antibiotic-related gastrointestinal risk across individual agents and classes [27]. Consequently, grouping all antibiotic exposures into a single category in the present study may have obscured associations limited to particular antibiotic classes. Because antibiotic class was not collected, we were unable to examine such heterogeneity, which limits the biological interpretation of the observed null adjusted association.

These differences are important because antibiotic-related GI effects may also depend on the duration of exposure, baseline gut health, infection severity, and time since treatment. Therefore, the absence of an independent adjusted association in our study suggests that antibiotic exposure alone did not explain current symptom burden after accounting for stronger clinical and psychosocial predictors. However, limited statistical power for the adjusted analyses means smaller independent associations cannot be excluded.

Prior GI diagnosis was the strongest independent predictor of GI symptoms in our sample (OR 2.50; B = 4.15), consistent with studies showing chronic GI conditions confer higher symptom burden and recurrence [28]. This strong association with current symptoms can also help explain why antibiotic use lost significance after adjustment: participants with underlying GI disease may be more likely to experience symptoms regardless of antibiotic exposure.

Higher perceived stress was associated with GI symptom burden in a graded pattern. This association may be linked to the brain-gut axis through changes in motility, visceral sensitivity, mucosal immune function, and pain perception [29]. The high prevalence of stress and its strong association with GI symptoms in our study suggest that psychological stress should be considered alongside medication exposure and infection history when interpreting symptom burden in this population.

The association between recent GI infection and higher symptom scores is consistent with a meta‑analysis reporting that more than 10% of patients developed IBS after infectious enteritis and that IBS risk was approximately fourfold higher following infection [30]. In our study, recent GI infection may have contributed directly to ongoing symptoms, or it may have triggered temporary post-infectious bowel disturbance. It may also explain part of the crude antibiotic association, because participants with recent infections are more likely to receive antibiotics and more likely to report GI symptoms. Therefore, infection is probably one of the main factors linking antibiotic use and symptoms before adjustment.

PPI use was independently associated with clinically relevant GI symptoms in the logistic model, although not with the continuous symptom score. This finding may mainly reflect confounding by indication: people who use PPIs are more likely to have reflux, dyspepsia, gastritis, or other upper-GI complaints [31], which increases their likelihood of reporting at least one moderate or severe GI symptom. A biological contribution is also possible because PPIs can alter the gut microbiome and have been linked to dysbiosis and enteric infection risk [32–35]. However, because the association appeared only for the binary outcome and not the continuous symptom score, it should be interpreted cautiously.

Female gender was associated with lower symptom burden in the adjusted models, which contrasts with prior literature that generally states women show higher IBS prevalence and increased risk of post-infectious IBS [30,36]. This unexpected result may be due to sample-specific factors, such as variation in previous GI diagnoses, healthcare-seeking behavior, smoking, stress reporting, symptom perception, or other unmeasured lifestyle and clinical factors.

## Conclusion

In this West Bank cross‑sectional study of 403 adults, antibiotic use in the prior three months was not independently associated with clinically relevant GI symptoms or symptom score after adjusting for covariates. Stronger independent correlates of GI symptom burden were a prior GI diagnosis, higher perceived stress (graded effect), and recent GI infection, while female gender was associated with lower symptom burden. Findings were robust across logistic, linear, and negative‑binomial models.

## Strengths and limitations

Strengths of this study include a sufficient sample size with no missing data, a GI symptom scale with good internal consistency (α = 0.861), detailed reporting of antibiotic use patterns, and multivariable plus sensitivity analyses that produced consistent findings in an understudied West Bank population. The study also collected detailed information on antibiotic exposure patterns, including number of courses, timing, duration, prescription source, adherence, use of multiple antibiotics, and probiotic advice. In addition, the use of multivariable logistic and linear regression models, together with a negative-binomial sensitivity analysis, allowed the robustness of the findings to be tested across different outcome definitions. The study also contributes evidence from an understudied West Bank population, where data on antibiotic use and GI symptom burden in the general community remain limited.

However, several limitations should be considered. The cross-sectional design prevents causal inference and does not allow the temporal sequence between antibiotic exposure and GI symptom onset to be established. The use of non-probability convenience sampling may have introduced selection bias, as individuals with internet access, greater engagement with social media, higher educational attainment, or greater interest in health-related topics may have been more likely to participate. Antibiotic exposure was based on self-report and was not independently verified using prescription, pharmacy, or medical records, which may have introduced recall bias, reporting bias, and exposure misclassification. Furthermore, participants were recruited exclusively from the West Bank; therefore, the findings may not be generalizable to Palestinians living in other geographic areas. The lack of detailed antibiotic exposure data (class, specific agent, dose, indication, and exact timing) prevented assessment of whether GI symptom burden differed according to specific antibiotic exposures and may have obscured class-specific associations. Residual confounding also remains possible because several potentially important factors were not measured in sufficient detail, including the indication for antibiotic treatment, severity of the underlying infection, broader dietary patterns, comorbid medical conditions, probiotic use, and healthcare access. These factors may be associated with antibiotic exposure and GI symptom burden and could therefore have influenced the adjusted estimates.

Although the GI symptom items were informed by established GSRS symptom domains and the nine-item scale demonstrated good internal consistency, the study-specific nine-item questionnaire did not undergo comprehensive psychometric validation. Therefore, measurement error and limitations in construct validity cannot be excluded. Moreover, the definition of clinically relevant GI symptoms was based on a study-specific severity threshold rather than a previously validated diagnostic cutoff; therefore, the binary outcome should be interpreted as an operational measure of symptom burden rather than a clinical diagnosis.

Finally, the sample size calculation was based on prevalence estimation rather than on statistical power for the multivariable regression analyses. Therefore, although the study included 403 participants and the adjusted models were successfully fitted, the study may have had limited power to detect small or moderate independent associations between antibiotic use and GI symptoms. Accordingly, the null association observed for antibiotic use should not be interpreted as definitive evidence of no effect. The confidence interval around the adjusted estimate (AOR = 1.06, 95% CI 0.69–1.65) indicates that potentially meaningful associations cannot be entirely excluded.

Future prospective studies with clinical verification, microbiological data, and more detailed antibiotic exposure assessment are needed to clarify causal pathways between antibiotic use, infection, stress, and GI symptom burden.

## Supporting information

S1 File Study questionnaire used to assess antibiotic exposure, clinical characteristics, and gastrointestinal symptoms. (DOCX)
