# Supplementary material for: Antibiotic use and gastrointestinal symptoms among adults in the West Bank, Palestine: A cross-sectional study
Source: PLoS One. 2026 Nov 13;21(11):e0353315. doi: 10.1371/journal.pone.0353315 (PMC13766244; doi:10.1371/journal.pone.0353315)
Supplement: Supplementary file 1 [file pone.0353315.s001.docx]

**S1 File. Study questionnaire**

**المعلومات العامة**

**١. العمر_______**

**٢. الجنس**

*حدد دائرة واحدة فقط*

○ ذكر

○ أنثى

**٣. مكان السكن**

*حدد دائرة واحدة فقط*

○ مدينة

○ قرية

○ مخيم

**٤. المستوى التعليمي**

*حدد دائرة واحدة فقط*

○ ثانوي أو أقل

○ دبلوم

○ بكالوريوس

○ دراسات عليا

**٥. الحالة الاجتماعية**

*حدد دائرة واحدة فقط*

○ متزوج/ة

○ غير متزوج/ة

○ مطلق/ة ، أرمل/ة

**٦. الحالة الوظيفية**

*حدد دائرة واحدة فقط*

○ طالب

○ موظف

○ عمل خاص

○ عاطل عن العمل/ متقاعد

**٧. متوسط الدخل الشهري**

*حدد دائرة واحدة فقط*

○ اقل من 1500 شيكل

○ 1500-3000 شيكل

○ 3000-5000 شيكل

○ اكثر من 5000 شيكل

**استخدام المضادات الحيوية**

**٨. هل استخدمت مضادات حيوية خلال آخر 3 أشهر؟**

*حدد دائرة واحدة فقط*

○ نعم

○ لا

*التخطي إلى السؤال ١٨*

**٩. كم مرة استخدمت المضادات الحيوية خلال آخر 3 أشهر؟**

*حدد دائرة واحدة فقط*

○ مرة واحدة

○ مرتين

○ ثلاث مرات

○ أربع مرات أو أكثر

**١٠. متى كان آخر استخدام للمضاد الحيوي؟**

*حدد دائرة واحدة فقط*

○ خلال آخر7 أيام

○ خلال آخر 8-30 يوم

○ خلال آخر 1-3 أشهر

**١١. ما مدة آخر كورس مضاد حيوي استخدمته؟**

*حدد دائرة واحدة فقط*

○ اقل من 5 ايام

○ 5-7 ايام

○ أكثر من 7 ايام

○ لا أتذكر

**١٢. هل كان استخدام آخر مضاد الحيوي بوصفة طبية من الطبيب؟**

*حدد دائرة واحدة فقط*

○ نعم

○ لا

**١٣. هل اكملت آخر كورس للمضاد الحيوي كما وصف لك؟**

*حدد دائرة واحدة فقط*

○ نعم بالكامل

○ جزئيًا

○ لا

○ بدون وصفة طبية

**١٤. خلال آخر مرة استخدمت فيها مضادًا حيويًا، هل تم وصف أو استخدام أكثر من نوع مضاد حيوي في نفس الفترة العلاجية (في نفس المرض أو العدوى)؟**

*حدد دائرة واحدة فقط*

○ نعم

○ لا

○ لا اتذكر

**١٥. اذا كانت الاجابة نعم، كم عدد المضادات الحيوية التي استخدمتها في آخر كورس؟**

*حدد دائرة واحدة فقط*

○ اثنان

○ ثلاثة

○ أكثر من ثلاثة

○ لا اتذكر

○ لم أستخدم اكثر من نوع مضاد حيوي في نفس الفترة

**١٦. هل استخدمت أيًّا مما يلي أثناء أو بعد المضاد الحيوي؟ يمكنك اختيار اكثر من اجابة**

*حدد كل الإجابات الملائمة*

□ بروبيوتيك

□ زبادي/أطعمة مخمّرة بشكل منتظم

□ أدوية للمعدة أو الحموضة

□ لا شيء مما سبق

**١٧. هل سبق أن نصحك الطبيب باستخدام البروبيوتيك مع المضاد الحيوي لتحسين صحة الجهاز الهضمي؟**

*حدد دائرة واحدة فقط*

○ نعم

○ لا

○ لا اتذكر

**أعراض الجهاز الهضمي**

**١٨. خلال آخر 4 أسابيع، ما شدة الأعراض التالية؟**

*حدد دائرة واحدة فقط في كل صف*

| **العرض** | **لا يوجد (0)** | **خفيف (1)** | **متوسط (2)** | **شديد (3)** |
| --- | --- | --- | --- | --- |
| ألم في أعلى البطن أو منتصفه | ○ | ○ | ○ | ○ |
| حرقة / رجوع حمض المعدة إلى الحلق / الفم | ○ | ○ | ○ | ○ |
| انتفاخ/ شعور مزعج بالامتلاء في البطن | ○ | ○ | ○ | ○ |
| زيادة الغازات او التجشؤ بشكل مزعج | ○ | ○ | ○ | ○ |
| إسهال | ○ | ○ | ○ | ○ |
| إمساك/ صعوبة في الإخراج | ○ | ○ | ○ | ○ |
| شعور بعدم الإفراغ الكامل بعد الاخراج | ○ | ○ | ○ | ○ |
| أصوات مزعجة / قرقرة في البطن | ○ | ○ | ○ | ○ |
| غثيان | ○ | ○ | ○ | ○ |

**١٩. كم مرة ظهرت هذه الأعراض الهضمية بشكل عام خلال آخر 4 أسابيع؟**

*حدد دائرة واحدة فقط*

○ لم تظهر

○ أقل من مرة أسبوعيًا

○ مرة أسبوعيًا 1-2

○ 3 مرات أسبوعيًا أو أكثر

○ بشكل شبه يومي

**٢٠. إلى أي مدى أثرت هذه الأعراض بشكل عام على نشاطك اليومي أو دراستك؟**

*حدد دائرة واحدة فقط*

○ لم تؤثر

○ اثرت قليلا

○ أثرت بدرجة متوسطة

○ اثرت كثيرا

**توقيت الأعراض**

**٢١. إذا كنت قد استخدمت مضادًا حيويًا، متى بدأت الأعراض الهضمية بالنسبة لآخر كورس مضاد حيوي؟**

*حدد دائرة واحدة فقط*

○ لم أعان من أعراض

○ كانت موجودة قبل استخدام المضاد الحيوي

○ بدأت أثناء استخدام المضادات الحيوية

○ بدأت خلال أسبوع بعد الانتهاء من المضاد الحيوي

○ بدأت بعد أكثر من أسبوع من الانتهاء من المضاد الحيوي

○ لا أستطيع التحديد

○ لم استخدم مضادًا حيويًا

**٢٢. هل تعتقد ان الأعراض ساءت بعد استخدام المضاد الحيوي؟**

*حدد دائرة واحدة فقط*

○ نعم

○ لا

○ لا أعلم

○ لم استخدم مضادًا حيويًا

○ لم اعاني من أعراض

**التاريخ المرضي والعوامل الصحية**

**٢٣. هل تم لديك تشخيص سابق بأي من الأمراض التالية؟**

*حدد دائرة واحدة فقط*

○ متلازمة القولون العصبي (Irritable Bowel Syndrome)

○ مرض الأمعاء الالتهابي (Inflammatory Bowel Disease)

○ قرحة المعدة أو الاثني عشر (Peptic Ulcer Disease)

○ ارتجاع مريئي

○ لا يوجد أي من هذه الأمراض

**٢٤. هل أصبت بعدوى أو نزلة معوية (قيء/إسهال) خلال آخر 4 أسابيع؟**

*حدد دائرة واحدة فقط*

○ نعم

○ لا

**٢٥. هل تستخدم حاليا او خلال آخر 4 اسابيع اي من الادوية التالية؟ يمكن اختيار اكثر من اجابة**

*حدد كل الإجابات الملائمة*

□ أدوية الحموضة للمعدة

□ مسكنات الألم (مثل: إيبوبروفين، باراسيتامول)

□ ملينات

□ مكملات الحديد

□ دواء منظم للسكري

□ لا شيء مما سبق

**نمط الحياة**

**٢٦. هل تدخن؟**

*حدد دائرة واحدة فقط*

○ نعم

○ لا

**٢٧. كم كوب قهوة تشرب يوميًا؟**

*حدد دائرة واحدة فقط*

○ لا أشرب القهوة

○ كوب واحد يوميًا

○ أكواب يوميًا 2-3

○ أكثر من 3 أكواب يوميًا

**٢٨. كم مرة تتناول الوجبات السريعة خلال الأسبوع؟**

*حدد دائرة واحدة فقط*

○ نادرًا

○ مرة في الأسبوع 1-2

○ 3 مرات أو أكثر في الأسبوع

**٢٩. كم تقّيم مستوى التوتر لديك خلال آخر 4 أسابيع؟**

*حدد دائرة واحدة فقط*

○ منخفض

○ متوسط

○ مرتفع

**المعرفة والسلوك**

**٣٠. هل تلاحظ أن بعض الأطعمة لديك تثير أعراض هضمية ؟**

*حدد دائرة واحدة فقط*

○ نعم

○ لا

○ لا أعلم

**٣١. هل تعتقد أن استخدام المضادات الحيوية قد يؤثر على صحة الجهاز الهضمي؟**

*حدد دائرة واحدة فقط*

○ نعم

○ لا

○ لا أعلم

**٣٢. هل سبق أن أُبلغت من طبيب أو صيدلي أن المضاد الحيوي قد يسبب أعراضًا هضمية؟**

*حدد دائرة واحدة فقط*

○ نعم

○ لا

○ لا أعلم

**English translation**

**General information**

**Age**

______________________________

**Sex**

| ○ Male | ○ Female |
| --- | --- |

**Place of residence**

| ○ City | ○ Village | ○ Refugee camp |
| --- | --- | --- |

**Educational level**

| ○ Secondary school or less | ○ Diploma | ○ Bachelor’s degree |
| --- | --- | --- |
| ○ Postgraduate studies |  |  |

**Marital status**

| ○ Married | ○ Unmarried | ○ Divorced / widowed |
| --- | --- | --- |

**Employment status**

| ○ Student | ○ Employed | ○ Private business |
| --- | --- | --- |
| ○ Unemployed / retired |  |  |

**Average monthly income**

| ○ Less than 1500 NIS | ○ 1500–3000 NIS | ○ 3000–5000 NIS |
| --- | --- | --- |
| ○ More than 5000 NIS |  |  |

**Antibiotic use**

**Have you used antibiotics during the past 3 months?**

| ○ Yes | ○ No |
| --- | --- |

**How many times have you used antibiotics during the past 3 months?**

| ○ Once | ○ Twice | ○ Three times |
| --- | --- | --- |
| ○ Four times or more |  |  |

**When was your most recent antibiotic use?**

| ○ During the past 7 days | ○ During the past 8–30 days | ○ During the past 1–3 months |
| --- | --- | --- |

**How long was the most recent course of antibiotics you used?**

| ○ Less than 5 days | ○ 5–7 days | ○ More than 7 days |
| --- | --- | --- |
| ○ I do not remember |  |  |

**Was your most recent antibiotic prescribed by a physician?**

| ○ Yes | ○ No |
| --- | --- |

**Did you complete your most recent antibiotic course as prescribed?**

| ○ Yes, completely | ○ Partially | ○ No |
| --- | --- | --- |
| ○ Without a prescription |  |  |

**During the most recent time you used an antibiotic, were you prescribed or did you use more than one antibiotic during the same treatment period (for the same illness or infection)?**

| ○ Yes | ○ No | ○ I do not remember |
| --- | --- | --- |

**If yes, how many antibiotics did you use during the most recent course?**

○ Two

○ Three

○ More than three

○ I do not remember

○ I did not use more than one antibiotic during the same period

**Did you use any of the following during or after the antibiotic? You may select more than one answer.**

□ Probiotics

□ Yogurt / fermented foods regularly

□ Stomach or acidity medications

□ None of the above

**Has a physician ever advised you to use probiotics with antibiotics to improve gastrointestinal health?**

| ○ Yes | ○ No | ○ I do not remember |
| --- | --- | --- |

**Gastrointestinal symptoms**

**During the past 4 weeks, how severe were the following symptoms?**

*Select one response in each row.*

| **Symptom** | **None (0)** | **Mild (1)** | **Moderate (2)** | **Severe (3)** |
| --- | --- | --- | --- | --- |
| Pain in the upper or middle abdomen | ○ | ○ | ○ | ○ |
| Heartburn / acid reflux into the throat / mouth | ○ | ○ | ○ | ○ |
| Bloating / uncomfortable feeling of fullness in the abdomen | ○ | ○ | ○ | ○ |
| Increased gas or bothersome belching | ○ | ○ | ○ | ○ |
| Diarrhea | ○ | ○ | ○ | ○ |
| Constipation / difficulty passing stool | ○ | ○ | ○ | ○ |
| Feeling of incomplete evacuation after passing stool | ○ | ○ | ○ | ○ |
| Bothersome abdominal sounds / rumbling | ○ | ○ | ○ | ○ |
| Nausea | ○ | ○ | ○ | ○ |

**How often did these gastrointestinal symptoms occur overall during the past 4 weeks?**

| ○ Did not occur | ○ Less than once weekly | ○ 1–2 times weekly |
| --- | --- | --- |
| ○ 3 or more times weekly | ○ Almost daily |  |

**To what extent did these symptoms overall affect your daily activities or studies?**

| ○ No effect | ○ Affected slightly | ○ Affected moderately |
| --- | --- | --- |
| ○ Affected greatly |  |  |

**Timing of symptoms**

**If you used an antibiotic, when did the gastrointestinal symptoms begin in relation to your most recent antibiotic course?**

○ I did not have symptoms

○ They were present before antibiotic use

○ They began during antibiotic use

○ They began within one week after completing the antibiotic

○ They began more than one week after completing the antibiotic

○ I cannot determine

○ I did not use an antibiotic

**Do you think the symptoms worsened after antibiotic use?**

| ○ Yes | ○ No | ○ I do not know |
| --- | --- | --- |
| ○ I did not use an antibiotic | ○ I did not have symptoms |  |

**Medical history and health factors**

**Have you previously been diagnosed with any of the following conditions?**

| ○ Irritable Bowel Syndrome | ○ Inflammatory Bowel Disease | ○ Peptic Ulcer Disease |
| --- | --- | --- |
| ○ Gastroesophageal reflux | ○ None of these conditions |  |

**Have you had a gastrointestinal infection or stomach flu (vomiting/diarrhea) during the past 4 weeks?**

| ○ Yes | ○ No |
| --- | --- |

**Are you currently using, or have you used during the past 4 weeks, any of the following medications? You may select more than one answer.**

□ Medications for stomach acidity

□ Painkillers (e.g., ibuprofen, paracetamol)

□ Laxatives

□ Iron supplements

□ Diabetes-regulating medication

□ None of the above

**Lifestyle**

**Do you smoke?**

| ○ Yes | ○ No |
| --- | --- |

**How many cups of coffee do you drink daily?**

| ○ I do not drink coffee | ○ One cup daily | ○ 2–3 cups daily |
| --- | --- | --- |
| ○ More than 3 cups daily |  |  |

**How often do you eat fast food during the week?**

| ○ Rarely | ○ 1–2 times per week | ○ 3 or more times per week |
| --- | --- | --- |

**How would you rate your stress level during the past 4 weeks?**

| ○ Low | ○ Moderate | ○ High |
| --- | --- | --- |

**Knowledge and behavior**

**Do you notice that some foods trigger gastrointestinal symptoms for you?**

| ○ Yes | ○ No | ○ I do not know |
| --- | --- | --- |

**Do you think antibiotic use may affect gastrointestinal health?**

| ○ Yes | ○ No | ○ I do not know |
| --- | --- | --- |

**Have you ever been informed by a physician or pharmacist that an antibiotic may cause gastrointestinal symptoms?**

| ○ Yes | ○ No | ○ I do not know |
| --- | --- | --- |
